# Food Security and Sustainability: Discussing the Four Pillars to Encompass Other Dimensions

**DOI:** 10.3390/foods10112732

**Published:** 2021-11-08

**Authors:** Raquel de Pinho Ferreira Guiné, Maria Lúcia de Jesus Pato, Cristina Amaro da Costa, Daniela de Vasconcelos Teixeira Aguiar da Costa, Paulo Barracosa Correia da Silva, Vítor João Pereira Domingues Martinho

**Affiliations:** Agricultural School (ESAV) and CERNAS-IPV Research Centre, Polytechnic Institute of Viseu (IPV), 3504-510 Viseu, Portugal; raquelguine@esav.ipv.pt (R.d.P.F.G.); mljesus@esav.ipv.pt (M.L.d.J.P.); amarocosta@esav.ipv.pt (C.A.d.C.); daniela@esav.ipv.pt (D.d.V.T.A.d.C.); pbarracosa@esav.ipv.pt (P.B.C.d.S.)

**Keywords:** FAOSTAT information, factor-cluster analyses, panel data approaches, quantile regressions

## Abstract

The unadjusted intake of food constitutes a real challenge for the several sustainability dimensions. In this perspective, the main objectives of this research are to characterise the current contexts of food security, its relationship with sustainability, and identify proposals and actions that may support the design of more adjusted policies in the future. In addition, it is intended to assess if the food security pillars properly address the sustainability goals and if the evolution of undernutrition is accompanied by sustainable frameworks. In this way, statistical information from the FAOSTAT database was considered for the several dimensions of food security over the period 2000–2020. These data were analysed through factor-cluster approaches and panel data methodologies, namely those related to quantile regressions. As main insights, we may refer that undernutrition is more impacted by the availability of food and nutrients and political stability than by the level of GDP—Gross Domestic Product (except for the extreme cases). This means that the level of development is not the primary explanation for the problems of nutrition. The main focus of the national and international policies must be to improve the agrifood supply chains and to support political stability, in order to mitigate undernutrition worldwide and ensure a global access to sustainable and healthy diets. In addition, it is suggested to rethink the four pillars of food security (availability, access, utilisation and stability), in order to encompass other dimensions, such as climate change.

## 1. Introduction

There is a significant relationship between the level of development of the countries and food insecurity contexts [1]. Food insecurity is also impacted by political stability and climate change [2].

Food security is a multidimensional concept characterised by four pillars related to availability, access, utilisation, and stability [3]. These dimensions are interrelated with, for example, agricultural modernisation [4], social capital [5], kitchen equipment [6], and worldwide shocks [7].

In a broader perspective, food security is interrelated with sustainable food and agricultural sectors, where the needs of present and future generations are met in commitment with the environmental, social, and economic dimensions [8]. The concepts of food security and sustainability are interrelated. The sustainability concept was considered by the international community associated with sustainable developments, according to which the societies evolve without compromising the future generations [9].

In fact, food security is already considered by the Sustainable Development Goals (SDGs), namely in the goal 2 for zero hunger [10]. Nonetheless, the pandemic context brought additional challenges for these world objectives increasing the problems associated with hunger worldwide, and it is expected that it will worsen malnutrition, namely among children [11]. On the other hand, the question here is if the food security pillars also specifically address the several dimensions of sustainability. The scientific literature shows that sustainability could be better encompassed by the four pillars of food security [9,12,13,14].

**Figure 1:**
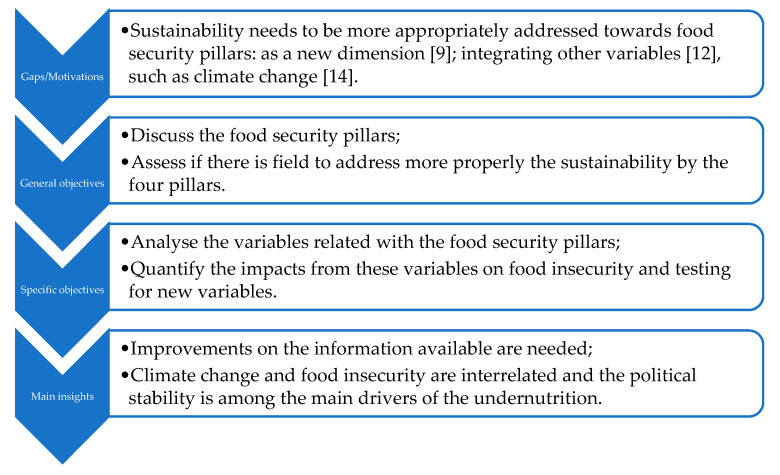
Gaps/motivations, general and specific objectives, and main insights.

From this perspective, the general and specific objectives are those presented in the following (summarised in Figure 1):

General objectives: This research aims to characterize the current context of world food security and its relationships with sustainability, through statistical information from FAOSTAT and panel data regressions (with quantile approaches), in order to suggest rethinking the four pillars to encompass other variables, such as climate change, and propose new policy instruments. The main focus is to highlight if sustainability has been sufficiently addressed by the food security pillars (namely testing for the impact of new variables, such as those related to climate change), considering that food security is already a target for the SDGs. In addition, it is the objective of this research to assess if food security has been achieved with sustainable approaches.

Specific objectives: Specifically, it is intended to characterise the variables related to the food security pillars over the period of 2000–2020 and quantify the impacts from these variables on food insecurity, identifying potentialities to encompass other variables (related to climate change, for example) in these security dimensions. This assessment allowed us to identify weaknesses in the database (there is a lack of statistical information for several countries and years), weaknesses in the group of variables considered for each pillar (it would be important to consider other variables, such as those related to the temperature change) and the main impacts from the four pillars’ variables on the food insecurity (with insights for the several stakeholders, namely for policymakers). This overview, considering the worldwide context and the approaches here carried out, has its novelty for the scientific community.

In summary, the literature shows that there are fields to be explored in these issues (“Sustainability needs to be more appropriately addressed towards food security pillars: as a new dimension [9]; integrating other variables [12], such as climate change [14].”). These fields were addressed through “—Analyse the variables related with the food security pillars; —Quantify the impacts from these variables on food insecurity and testing for new variables” that highlight “—Improvements on the information available are needed; —Climate change and food insecurity are interrelated and the political stability is among the main drivers of the undernutrition”.

The paper consists of six sections. After the introduction (Section 1), Section 2 contains a review of the concepts of food security and sustainability. The study design and materials are explained in Section 3, while Section 4 presents the main results of the study. To better assess the impacts of climate change on food security, Section 5 emphasises that the focus on food security should shift beyond the four pillars (availability, access, utilization, and stability) towards a vision that includes other sustainability variables. In Section 6, the discussion of the study is presented, as well as the main conclusions. In this last section, the study’s many results are emphasized, the theoretical, political, and practical contributions are highlighted, and the limitations of the study as well as areas for future research are presented.

A preliminary literature survey shows that there are few (or none) studies about the topic of food security and sustainability considering the methodologies hereby described, namely the quantile regressions with the approaches addressed, highlighting the novelty of this research and the potential to analyse deeper these domains.

## 2. Literature Survey

Food security is an old concern [15] that motivated international organizations, such as the Food and Agriculture Organization, to define it with the following four dimensions (or pillars): availability, accessibility, utilization, and stability [16]. Food security is part of the Sustainable Development Goals [11]. Nonetheless, it is accepted that sustainability needs to be more appropriately addressed in the pillars of food security, maybe as a new dimension [9], or integrating other variables [12], where the food crises [13] and climate change [14] have its importance.

Food security is interrelated with sustainability. However, the associated contexts are complex and uncertain [17], showing that these interrelationships deserve continuous attention from several stakeholders and organizations [18], including the subnational governments [19], and multidisciplinary approaches [20] for particular cases [21].

Agricultural practices have a great impact on the interrelationships between food security and sustainability [22] around the world [23] and for the more diverse contexts [24]; namely, those related with chemical approaches [25], water use [26], soil salinity [27], pests and diseases control [28], concentration/diversification of farming productions [29], intensification/extensification [30], and associated with management strategies [31]. The same happens with the different pressures over the agricultural land associated with, for example, the urbanization and ever-growing population [32]. In any case, the farming sector and the associated dimensions are determinants for sustainable food security [33], including animal welfare [34], and the new technologies open new opportunities [35], including for monitoring [36] and assessments [37]. The population growth is particularly worrying in countries such as India, for example [38], but other specific contexts also deserve special attention [39], including those from Africa [40].

For more sustainable food security, alternative sources of food supply, as edible insects, may be interesting solutions [41,42] in a framework of protein transition [43] and farm to fork perspective [44]. Urban agriculture may be another solution; however, in this case, additional studies are needed for a more complete assessment [45]. Farming fish is another possibility to achieve more sustainable food security [46], as are the home food gardens [47], revitalising local food systems [48], using insects as bioconverters of organic waste [49], obtaining protein from food waste [50], food forestry [51], natural food [52], better governance and conception [53], hydroponic farm [54], organic farming [55], crops resilience assessments [56], wild edible greens [57], food loss, and waste management [58].

Food security and sustainability are worldwide concerns, specifically for the policymakers [59]. In fact, the policies are crucial for sustainable food security [60]. In any case, the paradigms of food security often change with international crises, as happens currently with the COVID−19 pandemic [61], and these frameworks bring new challenges for the different stakeholders [62] to promote balanced and healthy diets [63].

In these interrelationships between food security and sustainability, the circular economy approaches may play a relevant role, namely through insect larvae that bioconvert food waste in animal feed [64].

## 3. Material and Methods

**Figure 2:**
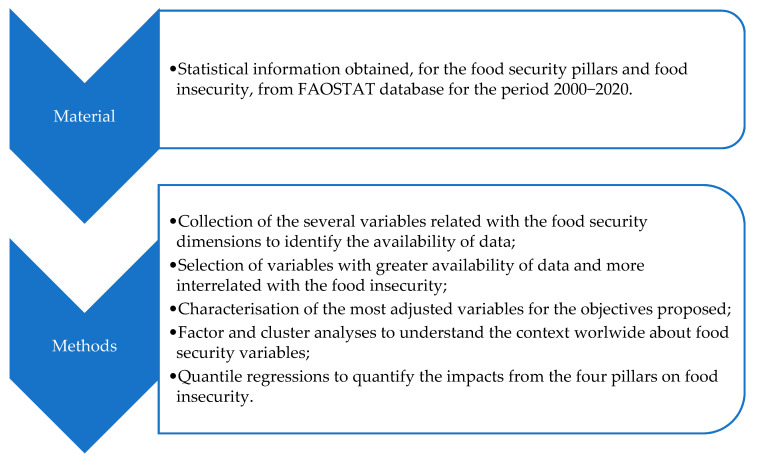
Schematic representation of the different steps under material and methods.

To better clarify the several steps of this section for material and methods, Figure 2 presents a schematic description of the various phases.

The quantile regressions are adjusted methodologies to deal with problems of outliers and non-normality in the statistical databases, and they were considered, for example, in the following studies related to the food security dimensions: factors affecting food diversity in Iran [65]; impacts on rice and wheat production in Pakistan [66]; implications from soil erosion on socio-economic domains in Malawi [67]; climate effects on the rice yield in India [68]; dietary deficiencies in the Indian context [69]; analysis of explanatory variables of calorie consumption in India [70]; food demand in Slovakia [71]; food insecurity in the population from the Afghanistan [72]; relationships between oil-export and food insecurity [73]; impacts from food insecurity on children [74]; food security in Nigerian urban households [75]; main drivers of healthy ageing in India [76]; effects from the internet use on the agricultural sustainability in China [77]; relationships among poverty and food security in Vietnam [78]; relations between religion and food security in India [79]; food security policies in India [80]; impacts from food prices on the nutrients intake in India [81]; interrelations between the efficiency of wheat and climate change in Pakistan [82]; crop diversity and welfare in Uganda [83]; drivers of food insecurity [84]; horticultural sector dynamics in Senegal [85]; and financial crisis and food supply in Mexico [86].

The statistical information was obtained from the FAOSTAT database [87] for the variables presented in Table 1 (and for the temperature change as an indicator for the climate changes) disaggregated across world countries and over the period of 2000–2020. The temperature change is correlated with the climate dimensions [88], being, in this way, an interesting indicator for the climate change contexts. Furthermore, it is one of the most straightforward quantitative indicators of climate change. These variables were grouped into five groups (level of development, nutritional and food availability, food insecurity, stability conditions, social and environmental conditions), following the suggestions from the statistical database. These five groups are relative to the four pillars of the food security concept (availability, access, utilization, stability) and a fifth set for food insecurity indicators (namely related to the number and prevalence of undernourishment and moderate and severe food insecurity). The four pillars were renamed as a suggestion to encompass other dimensions and to highlight the importance of rethinking these pillars and the food security dimensions. Some of these variables are presented in the database on average for periods of three years (Table 1). In these cases, to compare with other variables presented in annual values, it was considered the middle year as a reference. In addition, Table 1 shows that there is a lack of information for some countries and years. Due to this, the variables with lower observations were not considered in the analysis carried out in the following sections. The variables removed were: variable “Rail lines density (total route in km per 100 square km of land area)” from the group “level of development”; most variables related with the group “food insecurity”; and the variables “Percentage of children under 5 years affected by wasting (percent)” and “Prevalence of exclusive breastfeeding among infants 0–5 months of age” from the group “social and environmental conditions”.

Table 1 reveals that, on average, the world had, between 2000 and 2020, a Gross Domestic Product per capita (PPP, constant 2011) of 18905.540 I$, 2.753 km per 100 square km of land area for the rail density, 78.804 g/cap/day of average protein supply, 35.464 g/cap/day for average supply of protein of animal origin, 46.807 kcal/cap/day for the share of dietary supply derived from cereals, roots, and tubers, 14.322% of prevalence of undernourishment, 27.914% for cereal import dependency ratio, 28.430% for arable land equipped for irrigation, −0.032 for the political stability and absence of violence/terrorism (index) and several problems with social and environmental conditions. These results reveal that there is still much more to improve the nutritional conditions and the political stability, as well as the absence of violence/terrorism. In fact, Western Balkans and the European Union had, over the last years, values of 101.340 for average protein supply (g/cap/day), 57.300 average supply of protein of animal origin (g/cap/day) and 0.550 for political stability and absence of violence/terrorism (index), highlighting the way that must be run by some parts of the world in these domains [89].

In the different groups with the remaining variables, after the first selection identified before, the results presented in Table A1 (Appendix A) were considered to select the most representative variables in each group. Considering the great number of variables available in the database for four pillars, the correlation matrix brings insights that support the selection of the correlated variables. These are important findings to build the models for the regression approaches. These results were obtained following Stata procedures [90,91,92,93,94,95] for pairwise correlation matrices.

Table A1 (Appendix A) shows that there are positive and relatively strong correlations between the GDP (PPP, constant 2011 international $) and the supply of protein, political stability and access of the population to drinking water and sanitation services. In turn, there are negative and relatively strong relationships among the GDP and share of dietary energy supply from vegetables, percentage of children under 5 years of age who are stunted, and prevalence of anaemia among women of reproductive age (15–49 years). In addition, for the prevalence of undernourishment (percent), there are negative and relatively strong correlations with the GDP and the variables related to the “nutritional and food availability” group, with the exception of the variable “share of dietary energy supply derived from cereals, roots and tubers”, for which there is a positive correlation. The prevalence of undernourishment is also negatively correlated with political stability and with the access of the population to drinking water and sanitation services.

Considering this assessment and the relevance of the variables for the objectives proposed, the following variables were selected for each one of the groups considered: gross domestic product per capita, PPP, dissemination (constant 2011 international $) for the “level of development”, average protein supply (g/cap/day) (3-year average) for the group “nutritional and food availability”, prevalence of undernourishment (percent) (3-year average) for the “food insecurity”, political stability and absence of violence/terrorism (index) for “stability conditions” and percentage of population using safely managed drinking water services (percent) for “social and environmental conditions”. It could be interesting to consider also the prevalence of severe and moderate food insecurity, however, the limited availability of data for these variables hampers its consideration in regressions with panel data. 1. In fact, it is not possible to consider these variables with around 500 observations (or less) as shows Table 1, when the “Prevalence of undernourishment (percent) (3-year average)”, for example, has more than 2000 observations. In any case, for the cases (countries and years) where the observations are coincident the “Prevalence of undernourishment (percent) (3-year average)” (variable used by us) is strongly correlated (Table 2 obtained following Stata software procedures) with the severe and moderate undernourishment (showing that it is indifferent to use any of these variables and that our variable represents well the world context of undernutrition).

**Figure 3:**
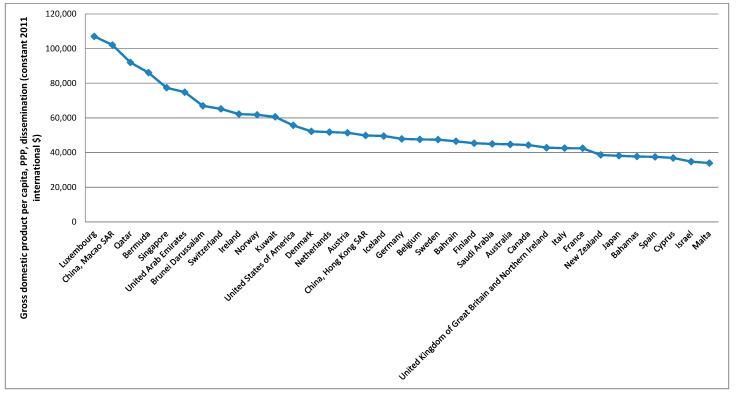
Top 35 countries for the level of development.

**Figure 4:**
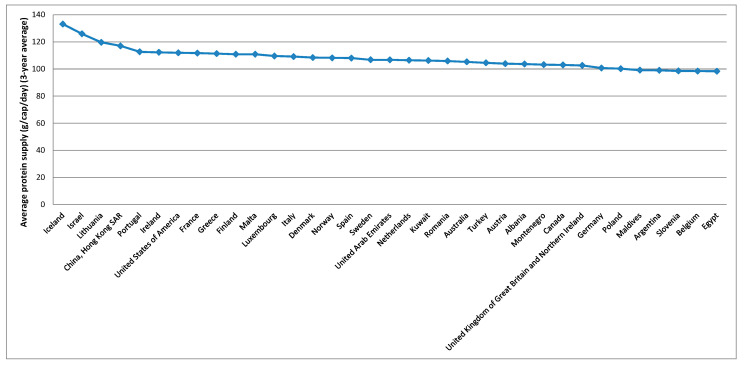
Top 35 countries for the nutritional and food availability.

**Figure 5:**
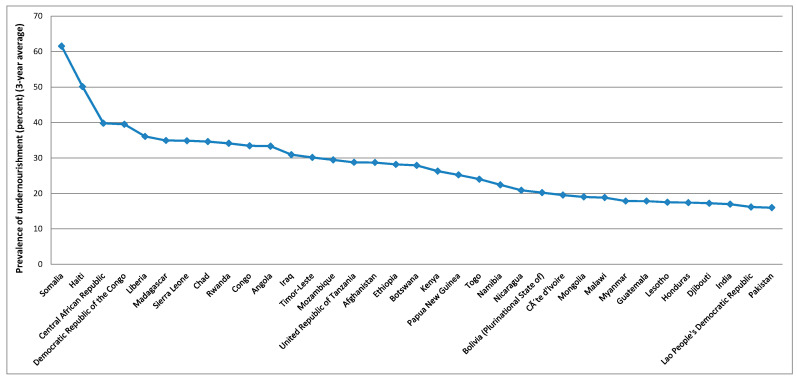
Top 35 countries for the level of food insecurity.

**Figure 6:**
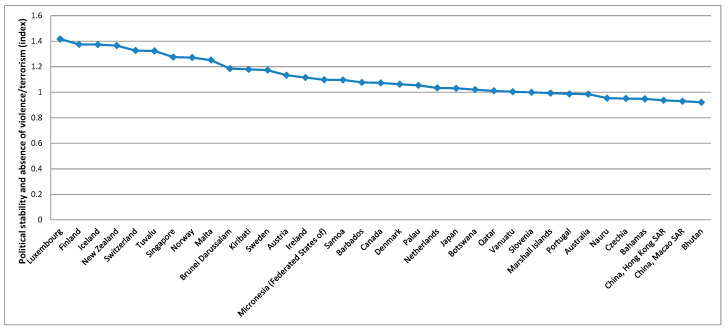
Top 35 countries for the stability conditions.

**Figure 7:**
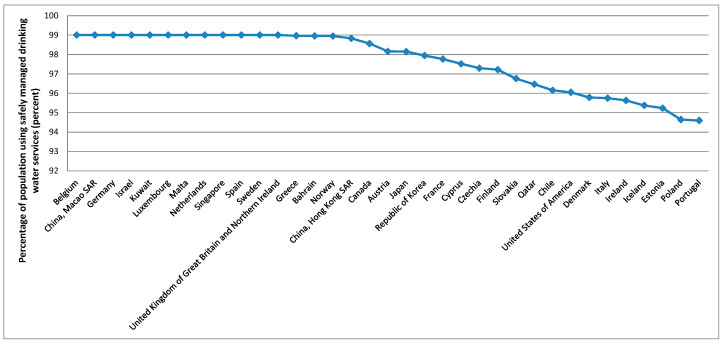
Top 35 countries for the social and environmental conditions.

To better analyse the evolution of these variables over the countries, averages over the period considered were calculated based on the summary statistics presented in Table 3, these being presented in Figure 3, Figure 4, Figure 5, Figure 6 and Figure 7.

Figure 3 shows that the five higher averages for the gross domestic product per capita, PPP, dissemination (constant 2011 international $), over the period considered, were verified in Luxembourg, China (Macao SAR), Qatar, Bermuda, and Singapore. For the average protein supply (g/cap/day) (3-year average), the five countries with higher averages are Iceland, Israel, Lithuania, China (Hong Kong SAR), and Portugal (Figure 4). Luxembourg, Ireland, Norway, USA and Denmark are among the countries with higher average GDP and average protein supply. These results confirm the findings described before for the correlations between the level of development and the nutrients supply [96]. The prevalence of undernourishment (percent) (3-year average) has higher averages in countries such as Somalia, Haiti, Central African Republic, Democratic Republic of the Congo, Liberia, and Madagascar, among others (Figure 5). Some of the countries with higher averages for the GDP and nutrients supply are also those with higher averages for the political stability and absence of violence/terrorism index (Figure 6) and percentage of population using safely managed drinking water services (Figure 7).

To better explore these data, a factor-cluster analysis following Stata procedures [93,94,95,97,98,99,100] was performed in the next section, to identify world clusters for variables that might be considered better drivers to implement and design joint policies. In addition, quantile regressions in panel data [93,101,102,103] were carried out considering a model based on the Verdoorn–Kaldor laws [104,105,106,107] and taking into account, for example, Martinho [108]. In the developments associated with the Verdoorn–Kaldor laws, the output growth is exogenous and promotes productivity growth, benefiting all the economy and society. Thus, following these developments, in this research, a model considering the output growth as an explanatory variable was considered. Due to the existence of outliers and lack of normality for some data, the quantile regression, based on the median estimation, was considered.

A factor analysis was carried out to obtain uncorrelated factors and to deal with problems of collinearity among the variables in the cluster assessment [100]. For that, a principal-component factors methodology with rotation was considered.

## 4. Results

**Figure 8:**
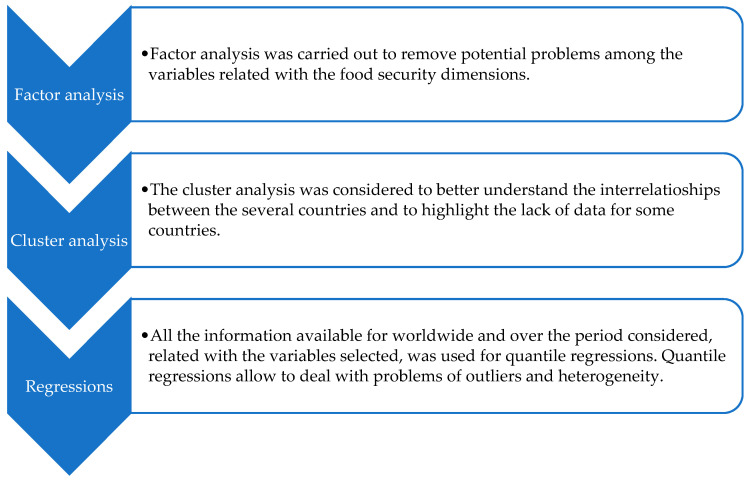
Summary of the several phases considered for the results.

Figure 8 summarises the different phases of this section regarding the results obtained with the variables selected for the dimensions associated with food security.

The five variables considered are correlated with factor1, explaining 65.8% of the variability (Table 4). The less relevant variables in the definition of factor 1 are the gross domestic product per capita, PPP, dissemination (constant 2011 international $) and political stability and absence of violence/terrorism (index). Inversely, the percentage of population using safely managed drinking water services is the most relevant. The results for the KMO in Table 4 show the sampling adequacy.

**Figure 9:**
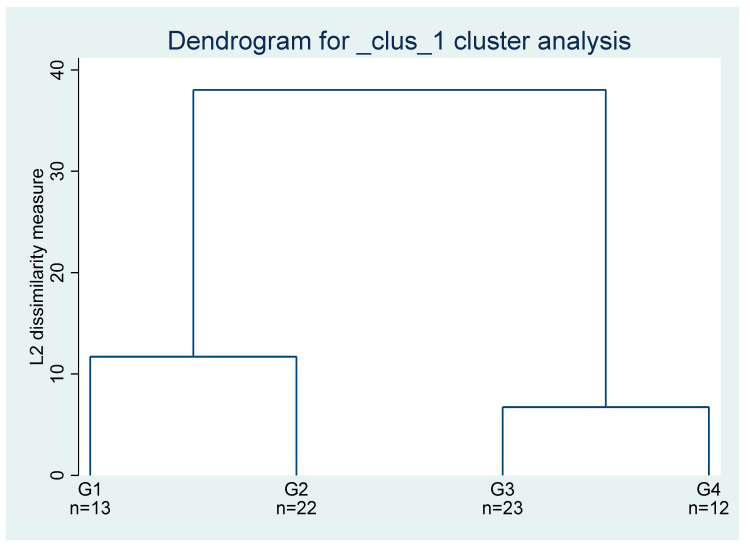
Results for cluster assessment, worldwide on average over the period of 2000–2020. Note: G1–G4 are branches (one for each cluster); and n is the number of observations in each branch.

Considering the information from Figure 9, four clusters were used for the cluster analysis whose results are presented in Table 5. Combining the results from Table 5 with the data analysis carried out previously, the cluster 1 identifies a set of countries where it is crucial to solve problems of food insecurity, namely those related to the prevalence of undernourishment (percent) (3-year average). For the countries in cluster 2 and cluster 4, there are also problems of undernourishment, but they are not so expressive as in the countries of cluster1. Japan and Republic of Korea appear in cluster 3 due to relatively lower scores for the protein supply, below Turkmenistan and Kazakhstan, respectively. In addition, the Republic of Korea has relatively lower averages for political stability and the absence of violence/terrorism (index). Table 5 is missing other world countries due to the lack of information for some variables. The countries for which there is lack of data for some variables are removed from the analysis, because the observations do not match when different variables are interrelated.

Before the panel data regressions, considering the characteristics of the sample, the data used for the variables were tested for normality, following, for example, Alejo et al. [103]. The results shown in Table 6 confirm the lack of normality. To deal with these statistical problems, quantile regressions with panel data were considered [109]. This approach is also adjusted to deal with the heterogeneity of the countries in the sample. For that, it was considered a model based on the Verdoorn–Kaldor laws, where the food insecurity variable (prevalence of undernourishment (percent) (3-year average)) was regressed with variables related to several dimensions of sustainability (level of development, nutritional and food availability, stability conditions and social and environmental conditions).

The results in Table 7 reveal that the undernourishment growth rates are mainly explained by the average protein supply (g/cap/day) (3-year average) growth rates and political stability, and absence of violence/terrorism (index) growth rates. Nonetheless, the political stability and absence of violence/terrorism improvement is not always synonymous of lower problems of undernutrition (as indicated by the sign found for the coefficient and the data analysis carried out before). On the other hand, the gross domestic product per capita, PPP, dissemination (constant 2011 international $) growth rates, and the percentage of the population using safely managed drinking water services (percent) growth rates are only statistically significant for the extreme cases (extreme quantiles). Furthermore, in the countries with lower growth rates for the prevalence of undernourishment (quantiles 0.10 and 0.20), the improvements in the GDP and drinking water services do not contribute to reducing the problems of undernutrition (the signals of the coefficients are positive).

## 5. Encompassing the Climate Change among the Pillars for a More Sustainable Food Security

**Figure 10:**
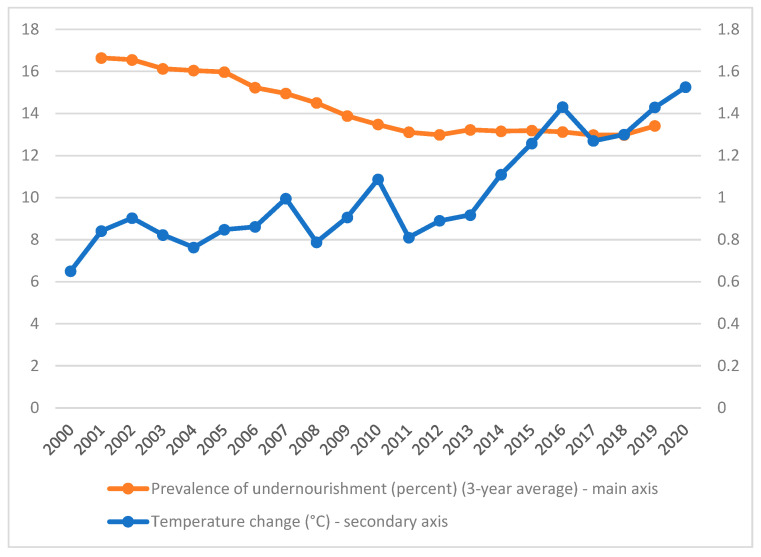
Benchmarking the prevalence of undernourishment and the temperature change, on average across the world over the period of 2000–2020.

Figure 10 reveals a negative relationship between the prevalence of undernourishment and the temperature change over the period considered in the world countries. In other words, the increase in temperature change verified since 2000 was accompanied by a decrease in the prevalence of undernourishment.

To better assess the impacts of climate change on food insecurity, several regressions were repeated, considering now the temperature change as an indicator for climate change (Table 8). The granger causality test for balanced panel data, using the Stata software, supports the temperature change as an explanatory variable. For the period of 2000–2020, the results for the variables previously considered are similar. The findings for the temperature change growth rates are, in general, statistically significant since the quantile is 0.30 and consistently negative. This means that increases in the temperature change growth rates that promote decreases in the prevalence of undernourishment growth rates, nonetheless with coefficients around −0.001 (when the temperature change increases 1% point, the undernutrition decreases 0.001% points).

These findings highlight that, despite the lower marginal impacts, the improvements in the mitigation of malnutrition in the world have been supported by unsustainable approaches, showing that there are here fields that deserve special attention by the national and international organizations.

To benchmark, shorter periods (2000–2013 and 2014–2020) were considered to carry out the regressions and the results are those presented in Table 9. These findings highlight, again, the relevance of the protein supply to reduce the prevalence of undernourishment. In addition, the results show that there are differences between the two shorter periods. For example, the importance of the political stability and the absence of violence to reduce the prevalence of undernourishment was more evident in the second (2014–2020) period (quantiles 0.50 and 0.60). The impacts from the temperature change were less visible in the second period; nonetheless, when these impacts are statistically significant, the coefficient is positive (quantiles 0.60 and 0.90), showing that in the most recent years, the impacts from climate change are more dramatic and related with increasing levels of undernourishment.

## 6. Discussion and Conclusions

The research here carried out intended to bring more insights into the world food security context and its relationship with sustainability. In other words, it was aimed to characterise the current context to understand if the four pillars associated with the concept of food security are enough and well-designed to deal with the present challenges, in a framework of sustainable development. To achieve these objectives, data from the FAOSTAT were considered, for the period of 2000–2020, that were assessed through, namely quantile regressions.

The food security concept is defined by four dimensions (availability, accessibility, utilization, and stability) and is interrelated with the Sustainable Development Goals. In fact, food security is part of the SDGs (goal 2 for zero hunger). Nonetheless, the question is if the sustainability dimensions are adequately considered by the food security pillars. The scientific literature highlights some concerns about these interrelationships and proposes either a new pillar [9] or considering other variables [12], namely those associated with climate change [14]. In addition, the several relationships between food security and sustainability are complex and need further multidisciplinary assessments. Agricultural practices, alternative sources of food supply, and public policies are determinants for more sustainable food security.

The data used shows that, worldwide over the period taken into account, the average protein supply was 78.804 g/cap/day, the average supply of protein of animal origin was 35.464 g/cap/day, the share of dietary supply derived from cereals, roots, and tubers accounted for 46.807 kcal/cap/day, and the political stability and absence of violence/terrorism (index) was −0.032. These results reveal that there is still much more to account for to improve the nutritional conditions and the political stability and absence of violence/terrorism. If we consider that the Western Balkans and the European Union had, over the period of 2016–2020, values of 101.340 for average protein supply (g/cap/day), 57.300 average supply of protein of animal origin (g/cap/day), and 0.550 for political stability and absence of violence/terrorism (index), there is a long way to run worldwide [89]. These findings highlight that a part of the world deals with problems of undernutrition while the other part has difficulties in reducing the prevalence of obesity. In any case, political stability seems to be one of the most important variables to improve the contexts of undernutrition.

The results from the regressions, for the period 2000–2020, show that undernutrition is principally impacted by the growth rates for average protein supply and for the political stability and absence of violence/terrorism (index). However, the political stability and absence of violence/terrorism are not enough to guarantee reductions in undernutrition. On the other hand, the economic and social conditions present evidences of statistically significance, namely in the countries with extreme values for undernourishment growth rates, but do not reduce the undernutrition in the countries with lower growth rates for the prevalence of undernourishment (first two quantiles). Finally, the indicator for the climate changes (temperature changes), in growth rates, contributed to reducing undernutrition growth rates. Considering that we used dynamic models with panel data, these results highlight, namely, that the undernutrition mitigation has been achieved through approaches associated with increases in the temperature. In fact, in specific contexts, adopting healthy diets may increase the environmental impacts [110]. In addition, the temperature changes impacts depend on local conditions [111]. With shorter periods (2000–2013 and 2014–2020), to benchmark, the findings highlight the importance of the political stability and absence of violence/terrorism to reduce the undernutrition and the negative impacts from the temperature change in the most recent years. Spearman’s rank correlation matrix [112] highlights the importance of the dependent variable considered for food insecurity in the regressions.

In terms of practical implications, it can be seen that the food security concept is part of the Sustainable Development Goals. However, sustainability needs to be more appropriately addressed inside the food security pillars, creating new dimensions or renaming the current pillars. This is important to give adjusted signs for several stakeholders. In terms of policy recommendations, it could be important to design instruments and measures that mitigate undernutrition through sustainable and more environmentally compatible practices. Regarding the limitations, the main constraints found to develop in this research were associated with the availability of statistical information. For future research, it is suggested to assess how the approaches used to mitigate undernutrition negatively impact the environment and contribute to climate change.

## Figures and Tables

**Table 1:** Summary statistics for several variables related to food security and sustainability, worldwide over the period 2000–2020.

Groups of Variables	Observations	Mean	Standard Deviation	Min	Max
**Level of development**					
Gross domestic product per capita, PPP, dissemination (constant 2011 international $)	3883	18,905.540	21029.290	630.700	161,971.000
Rail lines density (total route in km per 100 square km of land area)	1452	2.753	2.903	0.000	12.100
**Nutritional and food availability**					
Average dietary energy supply adequacy (percent) (3-year average)	3238	118.464	15.244	37.000	160.000
Average protein supply (g/cap/day) (3-year average)	2802	78.804	20.510	23.200	143.300
Average supply of protein of animal origin (g/cap/day) (3-year average)	2802	35.464	20.350	3.000	103.000
Average value of food production (constant 2004–2006 I$/cap) (3-year average)	3116	259.279	241.337	1.000	2425.000
Dietary energy supply used in the estimation of prevalence of undernourishment (kcal/cap/day) (3-year average)	3238	2793.793	467.732	903.000	3901.000
Share of dietary energy supply derived from cereals, roots and tubers (kcal/cap/day) (3-year average)	2802	46.807	14.640	8.000	83.000
**Food insecurity**					
Number of moderately or severely food insecure people (million) (3-year average)	483	8.809	14.699	0.100	116.000
Number of people undernourished (million) (3-year average)	1708	6.833	23.229	0.100	249.600
Number of severely food insecure people (million) (3-year average)	391	3.117	4.897	0.100	43.000
Prevalence of moderate or severe food insecurity in the total population (percent) (3-year average)	520	25.865	21.698	2.000	88.300
Prevalence of severe food insecurity in the total population (percent) (3-year average)	502	9.187	11.469	0.500	61.800
Prevalence of undernourishment (percent) (3-year average)	2047	14.322	11.401	2.500	71.300
**Stability conditions**					
Cereal import dependency ratio (percent) (3-year average)	2645	27.914	57.439	−342.500	100.000
Per capita food production variability (constant 2004–2006 thousand int$ per capita)	3090	12.323	13.048	0.200	107.700
Per capita food supply variability (kcal/cap/day)	3361	42.027	28.035	1.000	259.000
Percent of arable land equipped for irrigation (percent) (3-year average)	2797	28.430	31.498	0.000	100.000
Political stability and absence of violence/terrorism (index)	3486	−0.032	0.962	−3.180	1.760
Value of food imports in total merchandise exports (percent) (3-year average)	3227	41.389	89.288	1.000	1380.000
**Social and environmental conditions**					
Percentage of children under 5 years affected by wasting (percent)	616	6.136	4.382	0.000	21.300
Percentage of children under 5 years of age who are overweight (modelled estimates) (percent)	3047	7.049	4.467	0.700	29.300
Percentage of children under 5 years of age who are stunted (modelled estimates) (percent)	3047	21.918	14.624	1.200	62.300
Percentage of population using at least basic drinking water services (percent)	3803	85.024	17.929	18.100	99.000
Percentage of population using at least basic sanitation services (percent)	3802	72.130	29.831	2.800	99.000
Percentage of population using safely managed drinking water services (Percent)	2372	67.400	31.055	2.300	99.000
Percentage of population using safely managed sanitation services (Percent)	2384	53.409	29.468	2.100	99.000
Prevalence of anaemia among women of reproductive age (15–49 years)	3607	27.772	13.305	7.300	62.900
Prevalence of exclusive breastfeeding among infants 0–5 months of age	425	35.945	19.595	0.100	88.400
Prevalence of low birthweight (percent)	2255	10.312	4.920	2.400	36.200
Prevalence of obesity in the adult population (18 years and older)	3044	16.407	10.246	0.600	61.000

**Table 2:** Spearman’s rank correlation matrix for the several forms of prevalence of undernourishment worldwide over the period of 2000–2020.

	Prevalence of Moderate or Severe Food Insecurity in the Total Population (Percent) (3-Year Average)	Prevalence of Severe Food Insecurity in the Total Population (Percent) (3-Year Average)	Prevalence of Undernourishment (Percent) (3-Year Average)
Prevalence of moderate or severe food insecurity in the total population (percent) (3-year average)	1.000		
Prevalence of severe food insecurity in the total population (percent) (3-year average)	0.9200 *	1.000	
	0.000		
Prevalence of undernourishment (percent) (3-year average)	0.7917 *	0.7421 *	1.000
	0.000	0.000	

Note: *, statistically significant at 1%.

**Table 3:** Summary statistics for selected variables related to food security and sustainability worldwide on average over the period of 2000–2020.

Variables	Observations	Mean	Standard Deviation	Min	Max
Gross domestic product per capita, PPP, dissemination (constant 2011 international $)	187.000	18,809.910	20,670.980	817.519	107,015.200
Average protein supply (g/cap/day) (3-year average)	167.000	78.724	19.928	36.547	133.135
Prevalence of undernourishment (percent) (3-year average)	123.000	13.446	11.047	2.600	61.529
Political stability and absence of violence/terrorism (index)	186.000	−0.036	0.920	−2.473	1.417
Percentage of population using safely managed drinking water services (Percent)	115.000	67.658	30.806	5.600	99.000

**Table 4:** Results for rotated factor loadings (pattern matrix) and unique variances, worldwide on average over the period of 2000–2020.

Variables	Factor1	Uniqueness	KMO ^1^
Gross domestic product per capita, PPP, dissemination (constant 2011 international $)	0.738	0.455	0.695
Average protein supply (g/cap/day) (3-year average)	0.882	0.222	0.832
Prevalence of undernourishment (percent) (3-year average)	−0.850	0.278	0.715
Political stability and absence of violence/terrorism (index)	0.666	0.556	0.815
Percentage of population using safely managed drinking water services (Percent)	0.896	0.198	0.768
Overall			0.762

Note: ^1^ Kaiser–Meyer–Olkin measure of sampling adequacy.

**Table 5:** Identification of clusters, worldwide on average over the period of 2000–2020.

Clusters			
1	2	3	4
Afghanistan	Bangladesh	Albania	Algeria
Central African Republic	Cambodia	Belarus	Armenia
Chad	Colombia	Bosnia and Herzegovina	Azerbaijan
Congo	Ecuador	Brazil	Georgia
Côte d’Ivoire	Gambia	Bulgaria	Iran (Islamic Republic of)
Ethiopia	Ghana	Chile	Jordan
Iraq	Guatemala	China, Macao SAR	Mexico
Madagascar	Kiribati	Costa Rica	Morocco
Nigeria	Kyrgyzstan	Croatia	North Macedonia
Pakistan	Lao People’s Democratic Republic	Cyprus	Samoa
Rwanda	Lebanon	Estonia	Serbia
Sierra Leone	Lesotho	Japan	Ukraine
Togo	Mongolia	Kazakhstan	
	Myanmar	Kuwait	
	Nepal	Latvia	
	Nicaragua	Malaysia	
	Paraguay	Montenegro	
	Peru	Oman	
	Philippines	Republic of Korea	
	Sao Tome and Principe	Russian Federation	
	Suriname	Slovakia	
	Uzbekistan	Tunisia	
		Turkmenistan	

**Table 6:** Normality analysis, worldwide over the period of 2000–2020.

	Coefficient	z	*p* > |z|
Skewness_e	−0.271	−0.760	0.447
Kurtosis_e	4.186 *	2.720	0.006
Skewness_u	−0.862	−1.280	0.201
Kurtosis_u	2.994 *	2.560	0.010
Joint test for Normality on e: chi2(2) = 8.000 *, Prob > chi2 = 0.018			
Joint test for Normality on u: chi2(2) = 8.200 *, Prob > chi2 = 0.017			

Note: *, statistically significant at 5%.

**Table 7:** Results for panel data quantile regressions with the variables in growth rates, worldwide over the period of 2000–2020.

Prevalence of Undernourishment (Percent) (3-Year Average)	Coefficient	z	*p* > |z|
**Quantile 0.10**			
Gross domestic product per capita, PPP, dissemination (constant 2011 international $)	0.150 *	4.100	0.000
Average protein supply (g/cap/day) (3-year average)	−2.691 *	−49.820	0.000
Political stability and absence of violence/terrorism (index)	0.001 *	3.060	0.002
Percentage of population using safely managed drinking water services (Percent)	0.311 *	9.210	0.000
**Quantile 0.20**			
Gross domestic product per capita, PPP, dissemination (constant 2011 international $)	0.135 *	3.030	0.002
Average protein supply (g/cap/day) (3-year average)	−2.407 *	−14.720	0.000
Political stability and absence of violence/terrorism (index)	0.002 *	5.440	0.000
Percentage of population using safely managed drinking water services (Percent)	0.165 ***	1.670	0.094
**Quantile 0.30**			
Gross domestic product per capita, PPP, dissemination (constant 2011 international $)	−0.014	−0.440	0.657
Average protein supply (g/cap/day) (3-year average)	−2.275 *	−14.940	0.000
Political stability and absence of violence/terrorism (index)	0.003 *	19.480	0.000
Percentage of population using safely managed drinking water services (Percent)	−0.113 **	−2.400	0.017
**Quantile 0.40**			
Gross domestic product per capita, PPP, dissemination (constant 2011 international $)	−0.040	−1.140	0.256
Average protein supply (g/cap/day) (3-year average)	−2.259 *	−28.070	0.000
Political stability and absence of violence/terrorism (index)	0.003 *	15.510	0.000
Percentage of population using safely managed drinking water services (Percent)	−0.139 ***	−1.670	0.094
**Quantile 0.50**			
Gross domestic product per capita, PPP, dissemination (constant 2011 international $)	−0.005	−0.200	0.842
Average protein supply (g/cap/day) (3-year average)	−2.339 *	−26.550	0.000
Political stability and absence of violence/terrorism (index)	0.003 *	9.100	0.000
Percentage of population using safely managed drinking water services (Percent)	−0.063	−0.410	0.685
**Quantile 0.60**			
Gross domestic product per capita, PPP, dissemination (constant 2011 international $)	0.007	0.310	0.756
Average protein supply (g/cap/day) (3-year average)	−2.225 *	−23.670	0.000
Political stability and absence of violence/terrorism (index)	0.004 *	8.940	0.000
Percentage of population using safely managed drinking water services (Percent)	−0.100	−0.740	0.462
**Quantile 0.70**			
Gross domestic product per capita, PPP, dissemination (constant 2011 international $)	−0.005	−0.210	0.834
Average protein supply (g/cap/day) (3-year average)	−2.297 *	−16.500	0.000
Political stability and absence of violence/terrorism (index)	0.003 *	5.100	0.000
Percentage of population using safely managed drinking water services (Percent)	−0.021	−0.220	0.828
**Quantile 0.80**			
Gross domestic product per capita, PPP, dissemination (constant 2011 international $)	−0.045 **	−2.090	0.037
Average protein supply (g/cap/day) (3-year average)	−2.162 *	−24.610	0.000
Political stability and absence of violence/terrorism (index)	0.003 **	2.430	0.015
Percentage of population using safely managed drinking water services (Percent)	−0.225	−1.080	0.280
**Quantile 0.90**			
Gross domestic product per capita, PPP, dissemination (constant 2011 international $)	−0.119 *	−18.110	0.000
Average protein supply (g/cap/day) (3-year average)	−2.537 *	−168.980	0.000
Political stability and absence of violence/terrorism (index)	0.001 *	9.460	0.000
Percentage of population using safely managed drinking water services (Percent)	−0.094 *	−3.500	0.000
**Mean VIF = 1.030**			

Note: *, statistically significant at 1%; **, statistically significant at 5%; ***, statistically significant at 10%.

**Table 8:** Results for panel data quantile regressions with the variables in growth rates (encompassing climate change) worldwide over the period of 2000–2020.

Prevalence of Undernourishment (Percent) (3-Year Average)	Coefficient	z	*p* > |z|
**Quantile 0.10**			
Gross domestic product per capita, PPP, dissemination (constant 2011 international $)	0.077 **	2.090	0.037
Average protein supply (g/cap/day) (3-year average)	−2.674 *	−55.580	0.000
Political stability and absence of violence/terrorism (index)	0.001 *	4.080	0.000
Percentage of population using safely managed drinking water services (Percent)	0.337 *	3.740	0.000
Temperature change (°C)	0.000	0.160	0.875
**Quantile 0.20**			
Gross domestic product per capita, PPP, dissemination (constant 2011 international $)	0.077 *	3.080	0.002
Average protein supply (g/cap/day) (3-year average)	−2.718 *	−63.810	0.000
Political stability and absence of violence/terrorism (index)	0.002 *	5.370	0.000
Percentage of population using safely managed drinking water services (Percent)	0.152	1.640	0.101
Temperature change (°C)	0.000	0.810	0.420
**Quantile 0.30**			
Gross domestic product per capita, PPP, dissemination (constant 2011 international $)	−0.010	−0.450	0.652
Average protein supply (g/cap/day) (3-year average)	−2.280 *	−22.880	0.000
Political stability and absence of violence/terrorism (index)	0.003 *	12.740	0.000
Percentage of population using safely managed drinking water services (Percent)	−0.090 *	−2.760	0.006
Temperature change (°C)	−0.002 *	−6.060	0.000
**Quantile 0.40**			
Gross domestic product per capita, PPP, dissemination (constant 2011 international $)	−0.029	−0.800	0.424
Average protein supply (g/cap/day) (3-year average)	−2.243 *	−25.910	0.000
Political stability and absence of violence/terrorism (index)	0.003 *	13.550	0.000
Percentage of population using safely managed drinking water services (Percent)	−0.135 ***	−1.750	0.080
Temperature change (°C)	−0.001 *	−2.780	0.005
**Quantile 0.50**			
Gross domestic product per capita, PPP, dissemination (constant 2011 international $)	−0.021	−0.690	0.489
Average protein supply (g/cap/day) (3-year average)	−2.309 *	−28.610	0.000
Political stability and absence of violence/terrorism (index)	0.003 *	8.410	0.000
Percentage of population using safely managed drinking water services (Percent)	−0.165 ***	−1.710	0.087
Temperature change (°C)	−0.001 *	−3.670	0.000
**Quantile 0.60**			
Gross domestic product per capita, PPP, dissemination (constant 2011 international $)	−0.002	−0.090	0.927
Average protein supply (g/cap/day) (3-year average)	−2.302 *	−16.810	0.000
Political stability and absence of violence/terrorism (index)	0.003 *	3.070	0.002
Percentage of population using safely managed drinking water services (Percent)	0.117	0.880	0.379
Temperature change (°C)	−0.001 ***	−1.690	0.091
**Quantile 0.70**			
Gross domestic product per capita, PPP, dissemination (constant 2011 international $)	−0.034 *	−3.150	0.002
Average protein supply (g/cap/day) (3-year average)	−2.510 *	−24.620	0.000
Political stability and absence of violence/terrorism (index)	0.004 *	4.860	0.000
Percentage of population using safely managed drinking water services (Percent)	0.022	0.540	0.590
Temperature change (°C)	−0.001 *	−6.570	0.000
**Quantile 0.80**			
Gross domestic product per capita, PPP, dissemination (constant 2011 international $)	0.000	0.060	0.952
Average protein supply (g/cap/day) (3-year average)	−2.276 *	−31.300	0.000
Political stability and absence of violence/terrorism (index)	0.003 *	2.600	0.009
Percentage of population using safely managed drinking water services (Percent)	−0.398 *	−8.350	0.000
Temperature change (°C)	−0.001 **	−2.220	0.026
**Quantile 0.90**			
Gross domestic product per capita, PPP, dissemination (constant 2011 international $)	−0.131 *	−50.320	0.000
Average protein supply (g/cap/day) (3-year average)	−2.644 *	−195.770	0.000
Political stability and absence of violence/terrorism (index)	0.001 *	29.020	0.000
Percentage of population using safely managed drinking water services (Percent)	−0.160 *	−30.880	0.000
Temperature change (°C)	−0.000 *	−4.170	0.000
**Mean VIF = 1.030**			

Note: *, statistically significant at 1%; **, statistically significant at 5%; ***, statistically significant at 10%.

**Table 9:** Results for panel data quantile regressions with the variables in growth rates (encompassing climate change) worldwide over the periods of 2000–2013 and 2014–2020.

Prevalence of Undernourishment (Percent) (3-Year Average)	2000–2013			2014–2020		
	Coefficient	z	*p* > |z|	Coefficient	z	*p* > |z|
**Quantile 0.10**						
Gross domestic product per capita, PPP, dissemination (constant 2011 international $)	0.018	0.190	0.851	0.726 **	2.400	0.017
Average protein supply (g/cap/day) (3-year average)	−2.444 *	−42.520	0.000	−3.049 *	−2.660	0.008
Political stability and absence of violence/terrorism (index)	0.004 ***	1.710	0.087	0.007	0.440	0.658
Percentage of population using safely managed drinking water services (Percent)	0.336 *	2.990	0.003	1.596	1.110	0.267
Temperature change (°C)	0.000	0.210	0.836	−0.013	−0.500	0.615
**Quantile 0.20**						
Gross domestic product per capita, PPP, dissemination (constant 2011 international $)	0.007	0.160	0.877	0.430	1.640	0.100
Average protein supply (g/cap/day) (3-year average)	−2.527 *	−39.840	0.000	−3.248 *	−2.870	0.004
Political stability and absence of violence/terrorism (index)	0.003 *	4.690	0.000	0.010	1.270	0.205
Percentage of population using safely managed drinking water services (Percent)	−0.206 *	−5.320	0.000	3.117	1.430	0.153
Temperature change (°C)	0.000	−0.200	0.844	0.001	0.040	0.965
**Quantile 0.30**						
Gross domestic product per capita, PPP, dissemination (constant 2011 international $)	−0.147 *	−10.660	0.000	0.557 **	2.210	0.027
Average protein supply (g/cap/day) (3-year average)	−2.042 *	−28.890	0.000	−4.736 *	−4.110	0.000
Political stability and absence of violence/terrorism (index)	0.003 *	11.760	0.000	0.010	1.460	0.145
Percentage of population using safely managed drinking water services (Percent)	−0.012	−0.850	0.397	6.153 **	2.310	0.021
Temperature change (°C)	−0.002 *	−11.510	0.000	−0.011	−0.320	0.748
**Quantile 0.40**						
Gross domestic product per capita, PPP, dissemination (constant 2011 international $)	−0.049 **	−1.970	0.049	0.300	0.810	0.418
Average protein supply (g/cap/day) (3-year average)	−2.310 *	−28.600	0.000	−3.971 *	−3.500	0.000
Political stability and absence of violence/terrorism (index)	0.003 *	18.460	0.000	−0.009	−1.390	0.164
Percentage of population using safely managed drinking water services (Percent)	−0.101	−0.820	0.411	6.022 ***	1.720	0.085
Temperature change (°C)	−0.001 *	−2.580	0.010	−0.015	−0.480	0.631
**Quantile 0.50**						
Gross domestic product per capita, PPP, dissemination (constant 2011 international $)	−0.059 *	−3.180	0.001	−0.076	−0.800	0.422
Average protein supply (g/cap/day) (3-year average)	−2.387 *	−20.700	0.000	−1.758 *	−11.230	0.000
Political stability and absence of violence/terrorism (index)	0.003 *	6.670	0.000	−0.002 *	−4.270	0.000
Percentage of population using safely managed drinking water services (Percent)	−0.246 *	−3.630	0.000	0.482 *	3.530	0.000
Temperature change (°C)	−0.002 *	−31.530	0.000	0.005	0.450	0.654
**Quantile 0.60**						
Gross domestic product per capita, PPP, dissemination (constant 2011 international $)	−0.040	−1.500	0.132	−0.130	−1.410	0.158
Average protein supply (g/cap/day) (3-year average)	−2.526 *	−19.960	0.000	−2.682 *	−11.300	0.000
Political stability and absence of violence/terrorism (index)	0.004 *	8.610	0.000	−0.009 *	−3.040	0.002
Percentage of population using safely managed drinking water services (Percent)	0.006	0.040	0.971	0.776 *	4.590	0.000
Temperature change (°C)	−0.001 *	−2.980	0.003	0.012 *	5.760	0.000
**Quantile 0.70**						
Gross domestic product per capita, PPP, dissemination (constant 2011 international $)	−0.033 ***	−1.780	0.074	0.245	1.400	0.162
Average protein supply (g/cap/day) (3-year average)	−2.441 *	−17.780	0.000	−2.323 *	−3.620	0.000
Political stability and absence of violence/terrorism (index)	0.004 *	9.720	0.000	−0.008	−1.200	0.230
Percentage of population using safely managed drinking water services (Percent)	−0.046	−0.890	0.371	1.874 *	2.890	0.004
Temperature change (°C)	−0.001 *	−6.520	0.000	−0.003	−0.270	0.787
**Quantile 0.80**						
Gross domestic product per capita, PPP, dissemination (constant 2011 international $)	−0.041 **	−2.240	0.025	0.154	0.860	0.388
Average protein supply (g/cap/day) (3-year average)	−2.336 *	−34.910	0.000	−3.278 *	−3.660	0.000
Political stability and absence of violence/terrorism (index)	0.003 *	4.650	0.000	−0.002	−0.590	0.553
Percentage of population using safely managed drinking water services (Percent)	0.053	0.400	0.688	0.978	0.840	0.401
Temperature change (°C)	−0.002 *	−3.050	0.002	0.003	0.230	0.816
**Quantile 0.90**						
Gross domestic product per capita, PPP, dissemination (constant 2011 international $)	−0.044	−1.300	0.194	−0.065	−1.050	0.294
Average protein supply (g/cap/day) (3-year average)	−2.314 *	−22.000	0.000	−3.601 *	−12.630	0.000
Political stability and absence of violence/terrorism (index)	0.005 *	16.760	0.000	−0.001	−0.630	0.531
Percentage of population using safely managed drinking water services (Percent)	−0.212 **	−2.050	0.040	−1.012 *	−2.890	0.004
Temperature change (°C)	0.000	0.170	0.863	0.021 ***	1.740	0.083
**Mean VIF = 1.030**						

Note: *, statistically significant at 1%; **, statistically significant at 5%; ***, statistically significant at 10%.

## Data Availability

The data presented in this study are available on request from the corresponding author.

## Appendix A

**Table A1:** Pairwise correlation matrix between the variables related to the food security and sustainability worldwide over the period of 2000–2020.

	A	B	C	D	E	F	G	H	I	J	K	L	M	N	O	P	Q	R	S	T	U	V	W
A	1.000																						
B	0.4924 *	1.000																					
	(0.000)																						
C	0.6711 *	0.7896 *	1.000																				
	(0.000)	(0.000)																					
D	0.7497 *	0.6022 *	0.8918 *	1.000																			
	(0.000)	(0.000)	(0.000)																				
E	0.1955 *	0.3961 *	0.3968 *	0.4231 *	1.000																		
	(0.000)	(0.000)	(0.000)	(0.000)																			
F	0.6292 *	0.9521 *	0.8823 *	0.7537 *	0.4405 *	1.000																	
	(0.000)	(0.000)	(0.000)	(0.000)	(0.000)																		
G	−0.6645 *	−0.4572 *	−0.6577 *	−0.8441 *	−0.4024 *	−0.6219 *	1.000																
	(0.000)	(0.000)	(0.000)	(0.000)	(0.000)	(0.000)																	
H	−0.3220 *	−0.8478 *	−0.7302 *	−0.6002 *	−0.4028 *	−0.8871 *	0.4659 *	1.000															
	(0.000)	(0.000)	(0.000)	(0.000)	(0.000)	(0.000)	(0.000)																
I	−0.032	−0.1346 *	−0.1154 *	−0.1082 *	−0.3980 *	−0.1510 *	0.0507 *	0.027	1.000														
	(0.105)	(0.000)	(0.000)	(0.000)	(0.000)	(0.000)	(0.009)	(0.275)															
J	0.0909 *	0.2301 *	0.2487 *	0.2585 *	0.5546 *	0.2768 *	−0.2774 *	−0.2669 *	−0.4459 *	1.000													
	(0.000)	(0.000)	(0.000)	(0.000)	(0.000)	(0.000)	(0.000)	(0.000)	(0.000)														
K	−0.0393 *	−0.0845 *	−0.0627 *	−0.0491 *	−0.0945 *	−0.0698 *	0.014	0.0939 *	0.0650 *	0.008	1.000												
	(0.023)	(0.000)	(0.001)	(0.009)	(0.000)	(0.000)	(0.446)	(0.000)	(0.001)	(0.677)													
L	0.1909 *	0.0809 *	0.1254 *	0.0661 *	0.018	0.1118 *	−0.0397 *	−0.3093 *	0.2841 *	−0.0629 *	0.025	1.000											
	(0.000)	(0.000)	(0.000)	(0.001)	(0.342)	(0.000)	(0.042)	(0.000)	(0.000)	(0.001)	(0.202)												
M	0.5224 *	0.3307 *	0.5045 *	0.6388 *	0.2257 *	0.4623 *	−0.6006 *	−0.3959 *	0.039	0.1966 *	−0.0729 *	−0.0586 *	1.000										
	(0.000)	(0.000)	(0.000)	(0.000)	(0.000)	(0.000)	(0.000)	(0.000)	(0.053)	(0.000)	(0.000)	(0.003)											
N	−0.2110 *	−0.2366 *	−0.2111 *	−0.1593 *	−0.1905 *	−0.2559 *	0.0803 *	0.1231 *	0.2130 *	−0.0904 *	0.0670 *	−0.016	0.022	1.000									
	(0.000)	(0.000)	(0.000)	(0.000)	(0.000)	(0.000)	(0.000)	(0.000)	(0.000)	(0.000)	(0.000)	(0.409)	(0.219)										
0	0.1773 *	0.2945 *	0.4240 *	0.3412 *	0.2640 *	0.3978 *	−0.3242 *	−0.3133 *	−0.1089 *	0.2206 *	0.1737 *	0.1224 *	0.0605 *	−0.0718 *	1.000								
	(0.000)	(0.000)	(0.000)	(0.000)	(0.000)	(0.000)	(0.000)	(0.000)	(0.000)	(0.000)	(0.000)	(0.000)	(0.002)	(0.000)									
P	−0.5857 *	−0.5895 *	−0.6998 *	−0.7703 *	−0.3767 *	−0.7459 *	0.7829 *	0.6194 *	−0.005	−0.2767 *	−0.035	−0.2787 *	−0.4937 *	0.0590 *	−0.3245 *	1.000							
	(0.000)	(0.000)	(0.000)	(0.000)	(0.000)	(0.000)	(0.000)	(0.000)	(0.827)	(0.000)	(0.074)	(0.000)	(0.000)	(0.003)	(0.000)								
Q	0.5292 *	0.5721 *	0.6739 *	0.7133 *	0.2926 *	0.7096 *	−0.7021 *	−0.7030 *	−0.026	0.2225 *	−0.009	0.3283 *	0.4861 *	−0.1007 *	0.3804 *	−0.7775 *	1.000						
	(0.000)	(0.000)	(0.000)	(0.000)	(0.000)	(0.000)	(0.000)	(0.000)	(0.178)	(0.000)	(0.607)	(0.000)	(0.000)	(0.000)	(0.000)	(0.000)							
R	0.5875 *	0.5611 *	0.7268 *	0.7483 *	0.3185 *	0.7201 *	−0.7517 *	−0.6554 *	−0.0585 *	0.2391 *	0.013	0.3646 *	0.4581 *	−0.1524 *	0.4691 *	−0.7935 *	0.8803 *	1.000					
	(0.000)	(0.000)	(0.000)	(0.000)	(0.000)	(0.000)	(0.000)	(0.000)	(0.003)	(0.000)	(0.466)	(0.000)	(0.000)	(0.000)	(0.000)	(0.000)	(0.000)						
S	0.7033 *	0.6639 *	0.7982 *	0.8070 *	0.3286 *	0.7996 *	−0.7625 *	−0.6652 *	−0.047	0.1855 *	−0.0468 *	0.1635 *	0.5583 *	−0.3489 *	0.3454 *	−0.8148 *	0.8565 *	0.8691 *	1.000				
	(0.000)	(0.000)	(0.000)	(0.000)	(0.000)	(0.000)	(0.000)	(0.000)	(0.057)	(0.000)	(0.033)	(0.000)	(0.000)	(0.000)	(0.000)	(0.000)	(0.000)	(0.000)					
T	0.6890 *	0.5886 *	0.7427 *	0.7695 *	0.3229 *	0.7129 *	−0.7042 *	−0.4581 *	−0.0704 *	0.1756 *	−0.0882 *	0.1051 *	0.5376 *	−0.2559 *	0.0788 *	−0.6304 *	0.6457 *	0.7157 *	0.7704 *	1.000			
	(0.000)	(0.000)	(0.000)	(0.000)	(0.000)	(0.000)	(0.000)	(0.000)	(0.004)	(0.000)	(0.000)	(0.000)	(0.000)	(0.000)	(0.001)	(0.000)	(0.000)	(0.000)	(0.000)				
U	−0.5772 *	−0.4827 *	−0.6490 *	−0.7266 *	−0.4296 *	−0.6293 *	0.7274 *	0.4283 *	0.1163 *	−0.2737 *	0.0595 *	−0.1550 *	−0.5036 *	0.1357 *	−0.3943 *	0.5935 *	−0.6826 *	−0.7485 *	−0.7316 *	−0.6544 *	1.000		
	(0.000)	(0.000)	(0.000)	(0.000)	(0.000)	(0.000)	(0.000)	(0.000)	(0.000)	(0.000)	(0.001)	(0.000)	(0.000)	(0.000)	(0.000)	(0.000)	(0.000)	(0.000)	(0.000)	(0.000)			
V	−0.4089 *	−0.4257 *	−0.6067 *	−0.6238 *	−0.3829 *	−0.5464 *	0.6363 *	0.3434 *	0.2153 *	−0.2452 *	−0.0754 *	−0.1101 *	−0.3686 *	0.2607 *	−0.4712 *	0.5874 *	−0.4861 *	−0.6692 *	−0.6149 *	−0.4964 *	0.6058 *	1.000	
	(0.000)	(0.000)	(0.000)	(0.000)	(0.000)	(0.000)	(0.000)	(0.000)	(0.000)	(0.000)	(0.001)	(0.000)	(0.000)	(0.000)	(0.000)	(0.000)	(0.000)	(0.000)	(0.000)	(0.000)	(0.000)		
W	0.2944 *	0.5396 *	0.5513 *	0.5516 *	0.2140 *	0.6017 *	−0.6189 *	−0.4926 *	−0.014	0.2042 *	0.0425 *	0.2172 *	0.3923 *	0.0501 *	0.3431 *	−0.5739 *	0.5552 *	0.5537 *	0.4631 *	0.3194 *	−0.4476 *	−0.4938 *	1.000
	(0.000)	(0.000)	(0.000)	(0.000)	(0.000)	(0.000)	(0.000)	(0.000)	(0.490)	(0.000)	(0.025)	(0.000)	(0.000)	(0.008)	(0.000)	(0.000)	(0.000)	(0.000)	(0.000)	(0.000)	(0.000)	(0.000)	

Note: A, gross domestic product per capita, PPP, dissemination (constant 2011 international $); B, average dietary energy supply adequacy (percent) (3-year average); C, average protein supply (g/cap/day) (3-year average); D, average supply of protein of animal origin (g/cap/day) (3-year average); E, average value of food production (constant 2004–2006 I$/cap) (3-year average); F, dietary energy supply used in the estimation of prevalence of undernourishment (kcal/cap/day) (3-year average); G, share of dietary energy supply derived from cereals, roots and tubers (kcal/cap/day) (3-year average); H, prevalence of undernourishment (percent) (3-year average); I, cereal import dependency ratio (percent) (3-year average); J, per capita food production variability (constant 2004–2006 thousand int$ per capita); K, per capita food supply variability (kcal/cap/day); L, percent of arable land equipped for irrigation (percent) (3-year average); M, political stability and absence of violence/terrorism (index); N, value of food imports in total merchandise exports (percent) (3-year average); O, percentage of children under 5 years of age who are overweight (modelled estimates) (percent); P, percentage of children under 5 years of age who are stunted (modelled estimates) (percent); Q, percentage of population using at least basic drinking water services (percent); R, percentage of population using at least basic sanitation services (percent); S, percentage of population using safely managed drinking water services (Percent); T, percentage of population using safely managed sanitation services (Percent); U, prevalence of anaemia among women of reproductive age (15–49 years); V, prevalence of low birthweight (percent); W, prevalence of obesity in the adult population (18 years and older). *, statistically significant at 5%.
